# Bottom-up assembly of metallic germanium

**DOI:** 10.1038/srep12948

**Published:** 2015-08-10

**Authors:** Giordano Scappucci, Wolfgang M. Klesse, LaReine A. Yeoh, Damien J. Carter, Oliver Warschkow, Nigel A. Marks, David L. Jaeger, Giovanni Capellini, Michelle Y. Simmons, Alexander R. Hamilton

**Affiliations:** 1 School of Physics, University of New South Wales, Sydney, 2052, Australia.; 2 Department of Chemistry, Curtin University, Perth WA 6845, Australia.; 3 Nanochemistry Research Institute, Curtin University, Perth WA 6845, Australia.; 4 Centre for Quantum Computation and Communication Technology, School of Physics, The University of Sydney, Sydney NSW 2006, Australia.; 5 Department of Physics and Astronomy, Curtin University, Perth WA 6845, Australia.; 6 Department of Material Science and Engineering, University of North Texas, Denton, Texas 76209, United States.; 7 IHP, Im Technologiepark 25, 15236 Frankfurt (Oder), Germany.; 8 Dipartimento di Scienze, Università Roma Tre, Viale Marconi 446, 00146 Rome, Italy.; 9 Centre of Excellence for Quantum Computation and Communication Technology, School of Physics, University of New South Wales, Sydney, New South Wales 2052, Australia.

## Abstract

Extending chip performance beyond current limits of miniaturisation requires new materials and functionalities that integrate well with the silicon platform. Germanium fits these requirements and has been proposed as a high-mobility channel material, a light emitting medium in silicon-integrated lasers, and a plasmonic conductor for bio-sensing. Common to these diverse applications is the need for homogeneous, high electron densities in three-dimensions (3D). Here we use a bottom-up approach to demonstrate the 3D assembly of atomically sharp doping profiles in germanium by a repeated stacking of two-dimensional (2D) high-density phosphorus layers. This produces high-density (10^19^ to 10^20^ cm^−3^) low-resistivity (10^−4^Ω · cm) metallic germanium of precisely defined thickness, beyond the capabilities of diffusion-based doping technologies. We demonstrate that free electrons from distinct 2D dopant layers coalesce into a homogeneous 3D conductor using anisotropic quantum interference measurements, atom probe tomography, and density functional theory.

Doping germanium with homogeneous, free-electron concentrations well above the metal-insulator transition (*n*_3*D*_ = 10^17^ cm^−3^) enables low-resistivity source/drain extensions in high mobility transistors^1^,^2^, Ge-on-Si integrated lasers with maximal optical gain^3^,^4^,^5^, and plasma wavelengths (2–5 um) suitable for biological sensing^6^,^7^. Mainstream top-down implantation is inadequate for this purpose because enhanced dopant diffusion and the formation of neutral complexes create electrical deactivation and doping profiles that are broad and inhomogeneous^8^,^9^,^10^. Self-limiting surface reactions provide a promising alternative to control doping processes from the bottom-up^11^. This approach has produced monolayer-doped semiconductors with high-density, strongly confined, two-dimensional electron gases (2DEGs)^12^. Combinations of bottom-up and top-down approaches have been proposed^13^,^14^ to extend monolayer doping from 2D to 3D. While dopants are deposited in a single 2D layer, their distribution in 3D was obtained by thermal diffusion, with associated loss of atomic precision and profile homogeneity. Here we demonstrate an exclusively bottom-up approach to produce an effectively 3D doped, high-density, low-resistivity metallic germanium with a precisely defined thickness and doping profile.

## Results

### Sample growth and atom probe tomography characterisation

**Figure 1:**
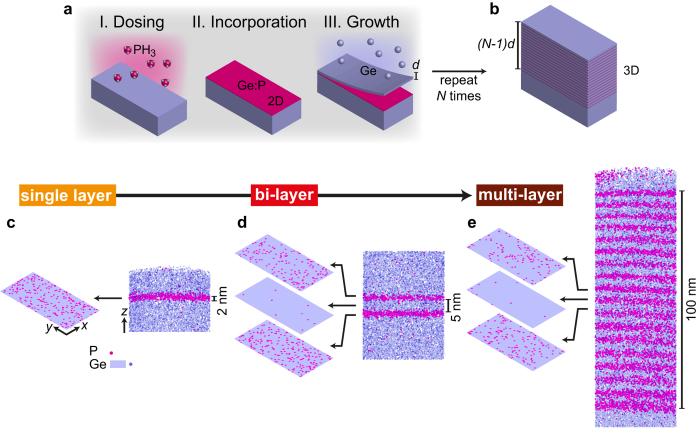
Three-dimensional assembly of atomically sharp doping profiles using a bottom-up approach. (**a**) Phosphorus doped layers in germanium are fabricated in ultra-high vacuum by adsorption of phosphine molecules (PH_3_) onto a clean Ge(001) surface, thermal incorporation of P atoms, and encapsulation under an epitaxial layer of germanium of thickness *d*. (**b**) Repetition of the sequence in (**a**) produces a highly doped Ge film of total thickness (*N* − 1)*d*. Pulsed laser atom probe tomography results from a (**c**) single layer, (**d**) bi-layer, and (**e**) multi-layer (18 layers) samples showing the cross-section distribution of dopant atoms.

The bottom-up assembly of metallic germanium is achieved by the repeated deposition of *N* nearly-identical phosphorus doped layers as illustrated in Fig. 1a. This approach preserves the vertical atomic-precision associated with monolayer doping and creates a homogeneous 3D system, provided the interlayer spacing is sufficiently small that the electrons can readily move between the layers. Each layer is prepared in a three-step process (Fig. 1a): (1) self-saturating chemisorption of phosphine molecules (PH_3_) onto a clean Ge surface^15^; (2) substitutional incorporation of P dopants into the Ge lattice by thermal annealing to provide a two-dimensional electron density *n*_2*D*_^12^; (3) encapsulation with Ge by molecular beam epitaxy to separate layers at a distance *d*. By stacking a large number of layers a precisely-doped slab of thickness *h* = (*N* − 1)*d* is produced. Using an inter-layer separation *d* comparable to the Bohr radius of phosphorus in germanium (≈8 nm), vertical electron delocalisation creates a homogeneous 3D system with a density *n*_3*D*_ = *Nn*_2*D*_/*h*. With current monolayer doping techniques^16^ achieving *n*_2*D*_ in the range of 10^13^ to 10^14^ cm^−2^ and with sub-10 nm layer separations, we can expect high 3D densities *n*_3*D*_ in the range of 10^19^ to 10^20^ cm^−3^. The transition from single 2D layers to an effectively 3D doped material is explored in this paper using a single-layer (Fig. 1d), a bi-layer (Fig. 1e), and a multi-layer sample (Fig. 1f) grown in ultra-high vacuum using the sequence described in Fig. 1a (Methods section). We use an interlayer separation of *d* ≈ 5.7 nm, which is less than the Bohr radius.

Three-dimensional atom probe tomography of these samples (Fig. 1c–e) shows dopant layers that are narrow and well separated. The dopant distribution of the single-layer sample has a full width at half maximum of 1.41 ± 0.05 nm. The P atoms are distributed randomly within the doping plane with a very high planar density of 1.44 × 10^14^ cm^−2^. A similar average width (1.4 ± 0.1 nm) and density (1.2 ± 0.3 × 10^14^ cm^−2^) is found for layers of the bi-layer sample. In the multi-layer sample the average width and density per layer are 2.0 ± 0.4 nm and (1.2 ± 0.3) × 10^14^ cm^−2^, respectively. There is a gradual increase in width by 0.06 nm from one layer to the next due to the accumulated thermal budget of the repeated deposition process (see Supplementary Section 1 for details). Crucially, we find that the inter-layer separation is preserved from layer to layer, averaging 5.65 nm with a variance of less than 0.05 nm. These metrics confirm that the vertical atomic-precision associated with monolayer doping is largely preserved when multiple layers are assembled into a larger stack.

### Electrical characterisation

By comparing atom probe tomography and Hall measurements (see Methods) we determine that approximately 26 to 44% of the dopants are electrically active. Despite incomplete activation, presumably due to the formation of P-P dimers^16^, the measured electronic planar densities *Nn*_2*D*_ are high at 6.3, 12, and 56 × 10^13^ cm^−2^ in the single layer, bi-layer and multi-layer (18-layer) sample, respectively. This corresponds to ultra-high 3D electronic densities of 4.5, 1.9, and 0.6 × 10^20^ cm^−3^. We measure exceptionally low resistivities of 2.0, 4.5, and 6.7 × 10^−4^ Ω ⋅ cm that are consistent with the resistivity vs. density dependence expected in heavily-doped bulk Ge^17^,^18^. This demonstrates that our bottom-up, “interface-free” doping technology achieves bulk-like resistivities in extremely thin (≈1.4 nm) doping profiles.

**Figure 2:**
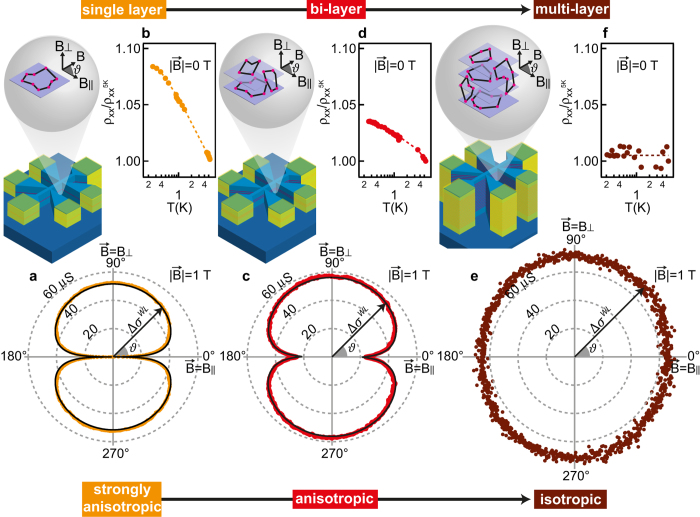
Evolution of the electronic system from 2D to 3D probed by electronic transport in different magnetic field orientations. (**a**,**c**,**e**) Polar plots of the size of the weak localization positive corrections to the magnetoconductivity 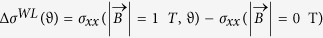 as a function of angle *ϑ* at a temperature of 200 mK for the single layer, bi-layer, and multi-layer samples, respectively; the angle *ϑ* is defined with respect to the dopant plane, with the components of magnetic field perpendicular and parallel to the plane of the dopant layers given by 
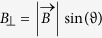
 and 
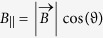
. Dashed grey lines are contours of constant Δ*σ*^*WL*^(*ϑ*). Black lines are theoretical fits to the data; (**b**,**d**,**f**) Temperature dependence of the zero magnetic field resistivity *ρ*_*xx*_ (normalized to its value 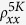 at *T* = 5 K) measured for the same samples (lines are a guide for the eye), showing how the characteristic log*T* behaviour for 2D systems weakens as the number of doping layers is increased. Illustrations of the Hall bars and weak localisation backscattered particle trajectories are also shown for the three samples.

Quantum interference measurements at cryogenic temperatures and in a vector magnetic field (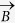) allow us to probe electron motion both within and between dopant layers, and observe the evolution from 2D towards a homogeneous 3D electronic system as the number of layers is increased. Figure 2 shows polar plots Δ*σ*^*WL*^(*ϑ*) of the weak localization (WL) positive corrections to the magnetoconductivity measured at constant field and variable angle *ϑ*. The single layer sample (Fig. 2a) exhibits a strong anisotropy with Δ*σ*^*WL*^(*ϑ*) at a maximum when 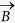 is perpendicular to the dopant plane. As the magnetic field is rotated, Δ*σ*^*WL*^(*ϑ*) collapses and is nearly negligible when 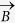 is parallel to the 2D layer. This finding is intuitive: the 2DEG is strongly confined in the vertical direction and hence the backscattered particle trajectories that give rise to WL are located within the 2D dopant plane. In the bi-layer sample (Fig. 2c), the anisotropy is still present but diminished. This indicates that in addition to motion within the layers, electrons are able to jump between them and form coherence interference loops that are affected by the parallel magnetic field. The two layers are strongly coupled despite the layer separation and effectively behave as a coherent 2D system of finite thickness. Quantitative analysis of the weak localisation yields the time scales associated with elastic scattering (*τ*_*e*_), interlayer tunneling (*τ*_*t*_), and dephasing (*τ*_*φ*_). The analysis shows 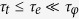 (see Supplementary Section 2 for details), confirming coherent tunnelling of electrons between layers over a timescale comparable to scattering off dopants within each layer. In the multi-layer sample, the polar plot (Fig. 2e) is nearly circular. The electron self-intersecting scattering paths are equally probable in all the three directions, supporting the presence of a homogeneous 3D system.

Temperature-dependent resistivity measurements at zero magnetic field provide additional confirmation of this 2D to 3D cross-over. Figure 2b,d,f show how the log*T* dependence characteristic of a 2D system^19^ is increasingly suppressed as layers are vertically stacked. Overall, this suppression of the temperature dependence and the progressive loss of anisotropy of the electron quantum interference reflect the loss of vertical confinement as electron motion approaches that of a 3D metallic conductor. Despite the atom probe imaging demonstrating that the sample comprises well-defined 2D layers, the strong inter-layer coupling means that the electrons see the sample as a bulk 3D material.

The thickness *h* of the conducting 2D systems in the single and bi-layer samples can be extracted by fitting Δ*σ*^*WL*^(*ϑ*) in Fig. 2a,c to a generalized angle-dependent Hikami-Larkin-Nagaoka expression^20^,^21^ (see Supplementary Section 3 for more details) that includes an additional dephasing rate due to the parallel magnetic field^22^,^23^. We obtain thicknesses of *h* = 1.49 ± 0.03 and 6.17 ± 0.05 nm for the single and bi-layer sample, respectively, in agreement with thicknesses of 1.41 ± 0.05 and 7.3 ± 0.2 nm obtained by atom probe tomography. This confirms the interpretation of the multi-layered doped region as a 3D space where electrons coming from 2D layered dopants can freely move, thus realizing a 3D metallic conductor.

## Discussion

**Figure 3:**
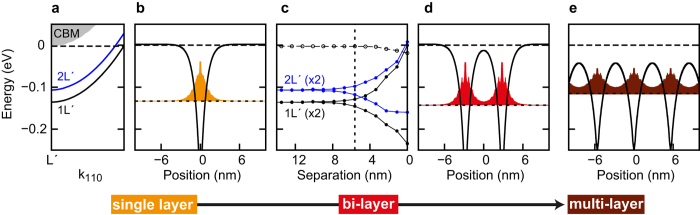
Density functional calculations of stacked dopant layers in germanium. (**a**) Band structure of a single phosphorus dopant layer (6.3 × 10^13^ cm^−2^) plotted from the 2DEG band minimum towards the zone centre. The bulk conduction band minimum (CBM) is indicated using grey shading. (**b**) Dopant potential and donor electron density for the same single layer structure. (**c**) Correlation of band energies for a pair of phosphorus dopant layers (both 6.3 × 10^13^ cm^−2^) as a function of separation. The Fermi energy is indicated by empty circles. For reference, the separation in the experimental bi-layer sample (5.7 nm) is indicated by a vertical dashed line. (**d**) The dopant potential and donor electron density for the same pair of dopant layers at the experimental separation. (**e**) Dopant potential and donor electron density for an infinite stack of dopant layers (each 3.1 × 10^13^ cm^−2^) with a spacing of 5.7 nm.

The critical role of the interlayer spacing *d* on the electronic transition from 2D to 3D behaviour is further explored using density functional theory in which the activated dopant densities are used as input parameters. A single phosphorus layer in germanium^24^ is characterized by a pair of valley-split bands (Fig. 3a, labelled 1L′ and 2L′), and a 2DEG density that is spatially confined to a width of ≈7.3 nm by the self-consistent doping potential (Fig. 3b). Figure 3c describes the evolution of the band minima of two dopant layers as the separation between the layers is reduced. At large separation, the two layers are effectively independent and the single layer band energies are preserved. At closer separation the band energies split due to coupling between the two layers. This commences at *d* ≈ 10 nm and is clearly established at 5.7 nm (dashed vertical line), the actual separation in our bi-layer sample. This coupling is also evident in the calculated electron density and doping potential (Fig. 3d) where the single layer densities and potentials are seen to overlap. In an infinite stack of layers (Fig. 3e) the overlap between the electron density (and the potential) of each layer is further enhanced, supporting the finding of strong 3D inter-layer coupling in our multi-layer sample.

In conclusion, our bottom-up approach to doping in Ge is capable of producing high electron densities (10^19^ to 10^20^ cm^−3^) and low-resistivity (10^−4^Ω ⋅ cm) metallic conductors of precisely defined thickness. As such, this technology has immediate relevance in electronics, photonics, and plasmonics, towards the development of high mobility transistors, industrially viable Si-integrated lasers, and mid-IR plasmonics bio-sensors, respectively. Finally, tunable doping at high densities also provides an ideal test-bed to clarify open questions on doping-induced bottom-up superconductivity in group-IV semiconductors, as recently proposed by Shim and Tahan^25^.

## Methods

### Sample preparation

All samples were fabricated in a customized ultra-high vacuum system (base pressure <5 × 10^−11^ mbar) comprising a MBE system (MBE Komponenten) for Ge deposition and an additional chamber for surface preparation and PH_3_ dosing. The chambers are connected via a UHV transfer tube. Ge(001) samples 2.5 × 10 mm^2^ in size were cleaved from a Sb doped Ge(001) 4 inch wafer (resistivity of 1–10 Ω ⋅ cm). Atomically flat, clean, and defect-free surfaces Ge(001) surfaces were prepared for all samples with the method detailed in Ref. 26. In brief, an *ex*-*situ* wet chemical treatment using HCl:H_2_O (36:100) and H_2_O_2_:H_2_O (7:100) is used to alternately strip and reform a germanium oxide passivation layer. This is subsequently removed *in*-*situ* by a flash-anneal at 760 °C, followed by a 25-nm Ge buffer layer growth by MBE at a rate of 0.015 nm/s and sample temperature of 400 °C. Prior to the first doping cycle step, the surface is flattened by a final thermal anneal at 760 °C. For P doping, all samples were saturation-dosed at room temperature with PH_3_ gas backfilling the UHV chamber at a pressure of ≈5 × 10^−11^ mbar via a leak valve. Thermal incorporation of P atoms into the Ge surface was obtained by increasing the sample temperature from room temperature to 400 °C at a rate of 1 °C/s. Epitaxial growth of Ge spacers 5.7-nm-thick was performed by MBE with the following growth temperature sequence. The first ≈0.7 nm of the Ge spacer are deposited at 400 °C, the following ≈2 nm at 250 °C, and the final 3 nm, again, at 400 °C. Between the first and second step of the sequence, the growth is interrupted to allow for sample cool-down at a rate of 1 °C/s. This sequence was engineered to minimize dopant diffusion and segregation whilst retaining a low-roughness surface at each doping cycle^27^. The deposition process ends for all samples with a 30-nm-thick Ge cap layer obtained by extending the duration of the third step in the growth temperature sequence.

### Pulsed laser atom probe tomography

After removal from UHV, all samples were cleaved and one portion was used to produce atom probe compatible specimens. All samples were coated with protective amorphous films of 25–60 nm Cr/20 nm Pt using a broad ion beam sputter system (Gatan 682 Precision Etching and Coating system). The coated samples were then transferred to a focused ion beam (FIB)/scanning electron microscope (SEM) dual-beam system (FEI Nova 200 Nanolab, Hillsboro, OR). Atom probe compatible needle specimens were prepared in the dual beam system using site specific lift-out techniques, then mounted to Si microtip posts (CAMECA Atom Probe Technology Center, Madison, WI) and finally annular milled. Pulsed laser atom probe tomography was performed at the University of North Texas Center for Advanced Research and Technology (CART) using a Local Electrode Atom Probe (LEAP) 3000X HR (CAMECA Atom Probe Technology Center, Madison, WI) laser pulsed local electrode atom probe with a reflectron lens. Samples were analysed at a base temperature of 30–50 K in laser pulsed field evaporation mode using a pulsed laser with a wavelength of 532 nm, pulse width of 10 ps, applied at a pulse frequency of 16 kHz, laser energy of 0.2–0.3 nJ and an evaporation rate of 0.001–0.005 ions/pulse. The resulting tomographic atom probe data was analysed using the atom probe reconstruction software, IVAS 3.6.1 (CAMECA Atom Probe Technology Center, Madison, WI). Reconstructions were correlated with Transmission Electron Microscopy and Secondary Ion Mass Spectrometry data (see Supplementary Section 1 for more details).

### Electrical characterization

Trench-isolated Hall bars structures to investigate the electrical properties of the doped layers were defined by a CHF_3_/CF_4_ based dry etch with thermally evaporated Al Ohmics connecting in parallel all multiple P layers of the doped stack. Electrical characterization at 4.2 K was performed using a dipstick in liquid helium equipped with a superconducting magnet providing a perpendicular magnetic field up to 2 T. Characterization of the device at lower temperatures between 0.2 and 5 K was performed in a cryogen-free dilution refrigerator, equipped with a triple axis vector magnet system (Leiden Cryogenics B.V.). This enables independent control of both perpendicular and parallel components of the magnetic field, with respect to the average dopant plane, and allows for magnetic field rotations to be performed at fixed field. The vector magnet was critical to performing reliable WL measurements to extract the 2D layer thickness since the large anisotropy in Δ*σ*^*WL*^ requires alignment of 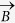 to better than 0.5 degrees with respect to the doping plane. For all measurements we used a four-probe setup using standard low frequency lock-in techniques and low injection currents (≈1 nA) to measure simultaneously the magnetic field dependence of the longitudinal *ρ*_*xx*_ and transverse *ρ*_*xy*_ components of the resistivity tensor, where *x* and *y* are, respectively, the directions parallel or perpendicular to the current flow in the Hall Bar. The longitudinal *σ*_*xx*_ and transverse *σ*_*xy*_ (Hall) conductivity were calculated from the measured resistivities via tensor inversion.

### Density functional theory calculations

DFT calculations on stacked phosphorus dopant layers in germanium were conducted using the SIESTA software^28^ and methods described for single Ge:P layers in Ref. 24. The DFT equations were solved using an atom-centered, double-numerical-plus-polarization (DNP) basis set and the local density approximation (LDA) with empirical on-site (+U) correction. The single and double dopant layer structures (Fig. 3a–d) were represented using highly elongated germanium super cells of 300 atomic layers which is sufficient to separate the dopant layers from their periodic repeats. For the repeated dopant layer stack (Fig. 3e) a much smaller unit cell of 40 layers was used in order to match the experimental layer separation of 5.7 nm. Phosphorus densities of 6.3 × 10^13^ cm^−2^ and 3.1 × 10^13^ cm^−2^ in the dopant plane were represented using the mixed-atom approach described in Ref. 24. All dopant atoms in the calculations are confined to a single atomic plane.

## Additional Information

**How to cite this article**: Scappucci, G. *et al*. Bottom-up assembly of metallic germanium. *Sci. Rep.* **5**, 12948; doi: 10.1038/srep12948 (2015).

## Supplementary Material

Supplementary Information

## References

[b1] Pillarisetty R. Academic and industry research progress in germanium nanodevices. Nature 479, 324–328 (2011). 22094692 10.1038/nature10678

[b2] Kamata Y. High-k/Ge MOSFETs for future nanoelectronics. Mater. Today 11, 30–38 (2008).

[b3] Liang D. & Bowers J. E. Recent progress in lasers on silicon. Nature Photon. 4, 511–517 (2010).

[b4] Liu J. F., Sun X. C., Camacho-Aguilera R., Kimerling L. C. & Michel J. Ge-on-Si laser operating at room temperature. Opt. Lett. 35, 679–681 (2010). 20195317 10.1364/OL.35.000679

[b5] Dutt B. . Roadmap to an Efficient Germanium-on-Silicon Laser: Strain vs. n-Type Doping. *Ieee Photonics J.* 4, 2002–2009 (2012).

[b6] Soref R. Mid-infrared photonics in silicon and germanium. *Nature Photon*. 4, 495–497 (2010).

[b7] Soref R., Hendrickson J. & Cleary J. W. Mid- to long-wavelength infrared plasmonic-photonics using heavily doped n-Ge/Ge and n-GeSn/GeSn heterostructures. Opt. Express 20, 3814–3824 (2012). 22418138 10.1364/OE.20.003814

[b8] Brotzmann S. & Bracht H. Intrinsic and extrinsic diffusion of phosphorus, arsenic, and antimony in germanium. J. Appl. Phys. 103, 033508 (2008).

[b9] Simoen E. & Vanhellemont J. On the diffusion and activation of ion-implanted n-type dopants in germanium. J. Appl. Phys. 106, 103516 (2009).

[b10] Chroneos A. & Bracht H. Diffusion of n-type dopants in germanium. Appl. Phys. Rev. 1, 011301 (2014).

[b11] Murota J., Sakuraba M. & Tillack B. Atomically controlled processing for group IV semiconductors by chemical vapor deposition. Jap. J. appl. Phys. 45, 6767–6785 (2006).

[b12] Scappucci G., Capellini G., Lee W. C. T. & Simmons M. Y. Ultradense phosphorus in germanium delta-doped layers. Appl. Phys. Lett. 94, 162106 (2009).

[b13] Ho J. C. . Controlled nanoscale doping of semiconductors via molecular monolayers. *Nature Mater*. 7, 62–67 (2008). 17994026 10.1038/nmat2058

[b14] Camacho-Aguilera R. E. . An electrically pumped germanium laser. Opt. Express 20, 11316–11320 (2012). 22565752 10.1364/OE.20.011316

[b15] Scappucci G. . n-Type Doping of Germanium from Phosphine: Early Stages Resolved at the Atomic Level. Phys. Rev. Lett. 109, 076101 (2012). 23006385 10.1103/PhysRevLett.109.076101

[b16] Mattoni G., Klesse W. M., Capellini G., Simmons M. Y. & Scappucci G. Phosphorus Molecules on Ge(001): A Playground for Controlled n-Doping of Germanium at High Densities. ACS Nano 7, 11310–11316 (2013). 24224765 10.1021/nn4051634

[b17] Spitzer W. G., Trumbore F. A. & Logan R. A. Properties of Heavily Doped N-Type Germanium. J. Appl. Phys. 32, 1822 (1961).

[b18] Weber B. . Ohm’s Law Survives to the Atomic Scale. Science 335, 64–67 (2012). 22223802 10.1126/science.1214319

[b19] Altshuler B. L., Aronov A. G. & Lee P. A. Interaction Effects in Disordered Fermi Systems in 2 Dimensions. Phys. Rev. Lett. 44, 1288–1291 (1980).

[b20] Hikami S., Larkin A. I. & Nagaoka Y. Spin-Orbit Interaction and Magnetoresistance in the 2 Dimensional Random System. Prog. Theor. Phys. 63, 707–710 (1980).

[b21] Shamim S. . Spontaneous Breaking of Time-Reversal Symmetry in Strongly Interacting Two-Dimensional Electron Layers in Silicon and Germanium. Phys. Rev. Lett. 112, 236602 (2014). 24972220 10.1103/PhysRevLett.112.236602

[b22] Mathur H. & Baranger H. U. Random Berry phase magnetoresistance as a probe of interface roughness in Si MOSFET’s. Phys. Rev. B 64, 235325 (2001).

[b23] Sullivan D. F., Kane B. E. & Thompson P. E. Weak localization thickness measurements of Si: P delta-layers. Appl. Phys. Lett. 85, 6362–6364 (2004).

[b24] Carter D. J. . Electronic structure of phosphorus and arsenic delta-doped germanium. Phys. Rev. B 88, 115203 (2013).

[b25] Shim Y. P. & Tahan C. Bottom-up superconducting and Josephson junction devices inside a group-IV semiconductor. Nature Commun. 5, 4225 (2014). 24985349 10.1038/ncomms5225

[b26] Klesse W. M., Scappucci G., Capellini G. & Simmons M. Y. Preparation of the Ge(001) surface towards fabrication of atomic-scale germanium devices. Nanotechnology 22, 145604 (2011). 21368353 10.1088/0957-4484/22/14/145604

[b27] Scappucci G., Capellini G., Klesse W. M. & Simmons M. Y. Phosphorus atomic layer doping of germanium by the stacking of multiple delta layers. Nanotechnology 22, 375203 (2011). 21857100 10.1088/0957-4484/22/37/375203

[b28] Soler J. M. . The Siesta method for ab initio order-N materials simulation. J. Phys.: Condens. Matter 14, 2745–2779 (2002).

