# Supplementary material for: Bottom-up assembly of metallic germanium
Source: Sci Rep. 2015 Aug 10;5:12948. doi: 10.1038/srep12948 (PMC4530340; doi:10.1038/srep12948)
Supplement: Supplementary Information [file srep12948-s1.pdf]

Supplementary Information for "Bottom-up assembly of metallic germanium"

Giordano Scappucci,[1,†] Wolfgang M. Klesse,[1] LaReine A. Yeoh,[1] Damien J. Carter,[2,3] Oliver Warschkow,[4] Nigel A. Marks,[5,3] David L. Jaeger,[6] Giovanni Capellini,[7,8] Michelle Y. Simmons,[1,9] and Alexander R. Hamilton[1]

[1]*School of Physics, University of New South Wales, Sydney, 2052, Australia*
[2]*Department of Chemistry, Curtin University, Perth WA 6845, Australia*
[3]*Nanochemistry Research Institute, Curtin University, Perth WA 6845, Australia*
[4]*Centre for Quantum Computation and Communication Technology, School of Physics, The University of Sydney, Sydney NSW 2006, Australia*
[5]*Department of Physics and Astronomy, Curtin University, Perth WA 6845, Australia*
[6]*Department of Material Science and Engineering, University of North Texas, Denton, Texas 76209, United States*
[7]*IHP, Im Technologiepark 25, 15236 Frankfurt (Oder), Germany*
[8]*Dipartimento di Scienze, Università Roma Tre, Viale Marconi 446, 00146 Rome, Italy*
[9]*Centre of Excellence for Quantum Computation and Communication Technology, School of Physics, University of New South Wales, Sydney, New South Wales 2052, Australia*

S1. ANALYSIS OF PHOSPHORUS DEPTH PROFILES OBTAINED BY ATOM PROBE TOMOGRAPHY

Atom probe tomography was used to create three-dimensional tomographic reconstructions of the single layer, bi-layer, and multi-layer samples which are shown in Fig. 1c-e of the main text. In Figures S1a-c atom probe tomography data is integrated into phosphorus atomic concentration profiles and compared to concentration profiles obtained by Secondary Ion Mass Spectrometry (SIMS). This comparison highlights the superior depth resolution of atom probe tomography to characterise the sharp dopant profiles in our samples. The lower resolution in the SIMS data is due to ion beam mixing from the sputtering process, which is known to lead to artificial peak broadening. The atom probe phosphorus profile for the single layer sample in Fig. S1a is slightly skewed to the left (i.e. towards the sample surface), which indicates that a degree of dopant segregation during growth is present. We fit this profile with an exponentially modified Gaussian (black line in Fig. S1a), which describes a diffusion-broadened profile with an exponential leading edge:[1]

$$N(x) = A\sqrt{\frac{\pi}{2}}\frac{w}{\lambda}\exp\left[\frac{(\mu - x)}{2} + \frac{1}{2}\left(\frac{w}{\lambda}\right)^2\right]\left[1 - \mathrm{erf}\frac{1}{\sqrt{2}}\left(\frac{(\mu - x)}{\lambda} + \frac{w}{\lambda}\right)\right] \tag{1}$$

We use as fitting parameters the $1/e$ decay length λ, and the amplitude A, position μ, and width w of the deconvolution Gaussian. We find $\lambda = 0.71 \pm 0.03$ nm, which corresponds to a segregation length scale of approximately five atomic layer in the germanium crystal. This shows that the growth process induces very little dopant segregation and hence the doping profiles are sharp at the atomic level.

The peaks in the bi-layer and multilayer concentration profiles (Fig. S1b, c) appear symmetric. The small inter-layer spacing limits phosphorus segregation in these samples and the peak shape is primarily determined by dopant diffusion from the accumulated thermal budget of the repeated deposition process. Hence, in these samples the doping profiles are better fitted with Gaussian functions (black lines in Fig. S1b and c).

	Single layer	Bi-layer(average)	Bi-layer (peak 2)	Bi-layer (peak 1)	Multi-layer (average)
FWHM (nm)	1.41 ± 0.05	1.4 ± 0.1	1.47 ± 0.05	1.32 ± 0.03	2.0 ± 0.4
Area (10^{14} cm^{-2})	1.44	1.2 ± 0.3	1.47	1	1.2 ± 0.3

Table S1. Analysis of phosphorus concentration profiles for single layer, bi-layer, and multilayer sample shown in Fig. S1a-c.

Table S1 summarises for all samples the key parameters reported in the main text. These parameters are the peak full width at half maximum (FWHM) and the area under the peak, obtained by numerical integration. In the single layer the FHWM is indicative of the dopant layer thickness h. We assume for FHWM in the single layer the same percentage error (3.6%) of the deconvoluted Gaussian FWHM obtained from fitting Eq. (1) to the data. In the bi-layer, the thickness is estimated by adding the finite average peak width to the interlayer separation,

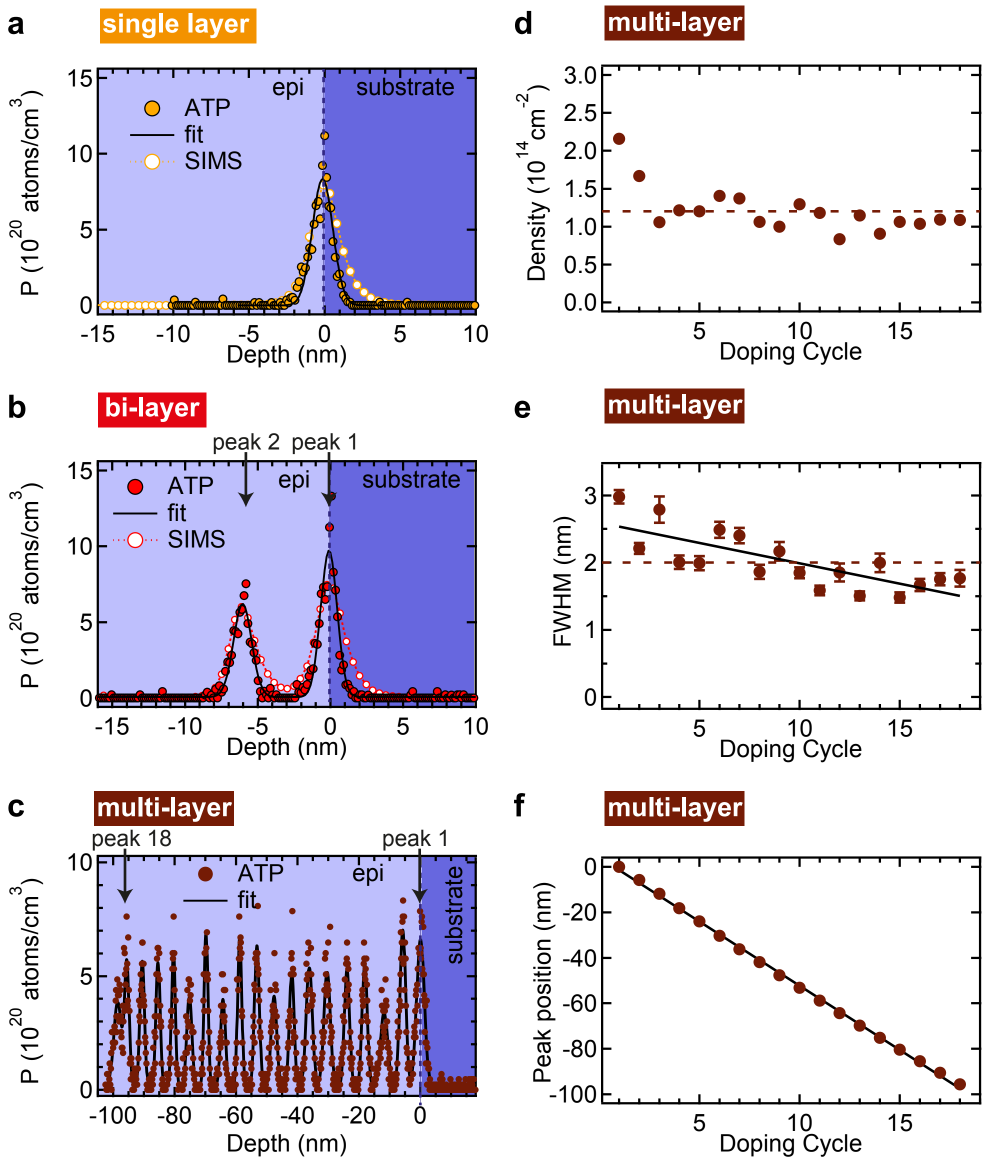

Figure S1: Analysis of atom probe tomography results. Phosphorus atomic concentration profiles from a (a) single layer, (b) bi-layer, and (c) multi-layer (18 layers) obtained by atom probe tomography (coloured circles) or SIMS (white circles) as a reference. Black lines are fit to the data. Analysis of individual peaks of the multi-layer sample showing: (d) planar doping density by integrating the area under each peak (dashed line is the average value); (e) full width at half maximum (FWHM) obtained by a Gaussian fit (dashed line is the average value);(f) peak position. Black lines in (e) and (f) are linear fits to the data from which we obtain the average FWHM broadening and inter-layer separation, respectively. In (d)-(f) the first doping cycle corresponds to the peak 1 in the concentration profile in (c).

$h = d + \text{FHWM} = 7.3 \pm 0.2$ nm. For all samples, the area under each peak is indicative of the planar P density per layer (n_P). The values achieved are comparable to previous studies of single monolayer doped Ge:P samples.[2]

In the multilayer sample, the average peak FWHM is increased to approximately 2 nm. To investigate the origin of this broadening, the FWHM for each layer are shown in Fig. S1e. The data shows that the width progressively decreases from the first deposited layer to the last. This can be understood in terms of an accumulated thermal budged. The first deposited layer is the broadest because it undergoes a small amount of thermal diffusion during the annealing and growth step of each subsequent doping cycle. The last deposited layer is the sharpest because experiences only a single thermal processing step. A linear fit of FWHM versus Doping Cycle quantifies this broadening as approximately 0.06 nm per doping cycle. Despite this broadening, the layers are still distinct and their separation preserved. This is illustrated in Fig. S1e, where the linear increase in the peak position with the doping cycle yields an inter-layer

separation value of 5.65 ± 0.05 nm, as reported in the main text.

S2. QUANTITATIVE ANALYSIS OF THE WEAK LOCALISATION FEATURE.

In this section we provide further details into our measurements of the magnetoconductivity $\sigma_{xx}(\vec{B})$ with orientation of the vector magnetic field perpendicular ($B_\perp$) or parallel ($B_\|$) with respect to the 2D doping layers. We perform a quantitative analysis of the weak localisation correction in perpendicular magnetic field to obtain the relevant timescales associated with electron transport, indicating strong electronic coupling between stacked dopant layers. Throughout the range of temperatures investigated, electron transport is in the diffusive regime with very short measured elastic scattering times τ_e of 0.9×10^{-14}, 1.0×10^{-14}, and 1.8×10^{-14} s in the single layer, bi-layer, and multi-layer sample, respectively.

Figure S2a, b, and c compare the conductivity σ_{xx} as a function of $B_\perp$ and $B_\|$ (black and grey curves, respectively) for the single layer, bi-layer, and multi-layer, respectively. In the single layer (Fig. S2a) weak localisation due to phase coherent backscattering reduces significantly the conductivity at zero magnetic field. The characteristic signature of weak localisation is a peaked positive magnetoconductance in perpendicular field, as $B_\perp$ threads flux through closed loops of particle backscattering trajectories and kills the weak localisation feature. In contrast, when the magnetic field is parallel to the 2D doping plane, very little flux is threaded through the particle trajectories, so almost no suppression of weak localisation is observed, and, consequently, the magnetic field dependence is weak. At $B = 1$ Tesla (circles in Fig. S2a) a strong anisotropy is established. This anisotropy is also evident in the polar plot in Fig 2b of the main text. In line with previous weak localisation measurements on Ge:P single layers,[3] we observe in parallel magnetic field a small negative weak anti-localization correction, due the local moments in the presence of strong Coulomb interactions.

In the bi-layer sample (Fig. S2b), the perpendicular field dependence resembles that of the single layer sample. In the parallel direction, there is now significant field dependence because $B_\|$ threads flux through particle backscattering trajectories formed in-between layers due to strong coupling. In this sample, the anisotropy at 1 Tesla is still present but diminished. In the multi-layer sample (Fig. S2c), the perpendicular and parallel magnetoconductance are seen to be almost overlapping, and hence the WL correction to the magnetoconductivity is nearly isotropic.

In Figure S2d we show the perpendicular field magnetoconductivity per layer $\sigma_{xx}(B_\perp)/N$ with $N = 1$, 2, and 18 in the single-layer, bi-layer and multilayer sample respectively. The measurements are over an extended range of magnetic fields to compare directly the magnitude of the WL correction across the three samples. We note a progressive suppression of the WL feature around zero magnetic field as the number of layers is increased, confirming the evolution of the electronic system from 2D to 3D. Note that if the layers were fully decoupled, all samples would show a similar dip in the magnetoconductance per layer. Associated with the dimensional cross-over is also the approximately twofold increase in electron mobility from the single-layer to the multi-layer. This is due to the population of 3D states extended in the vertical direction with reduced scattering from impurities within the doping planes.

The relevant timescales of electron transport in the single layer and an insight into electronic coupling in the bi-layer sample are obtained by fitting of the WL feature in perpendicular field. For all fits we have subtracted the classical correction to the Drude conductivity $\sigma_D(\mu B)^2$. In the single layer (Fig. S2e), the magnetoconductivity $\Delta\sigma_{xx} = \sigma_{xx}(B_\perp) - \sigma_{xx}(0)$ is fitted to a phenomenological generalized Hikami-Larkin-Nagaoka expression for the quantum interference correction to the conductivity:[3]

$$\Delta\sigma_{xx}(B_\perp) = \frac{\alpha e^2}{2\pi^2\hbar}\left[F\left(\frac{\hbar}{4eD\tau_\varphi B_\perp}\right) - F\left(\frac{\hbar}{4eD\tau_e B_\perp}\right)\right] - \frac{\beta e^2}{2\pi^2\hbar}F\left(\frac{\hbar}{4eD\tau_s B_\perp}\right) \tag{2}$$

In this expression α and β are positive constants close to unity, $F(x) = \digamma(0.5 + x) - ln(x)$ with $\digamma(x)$ being the digamma function, D is the two-dimensional diffusion constant, τ_s and τ_φ are the timescales characterising quasielastic spin scattering and dephasing, respectively. Excellent agreement between fit (black curve) and experimental data is obtained over the entire range of magnetic fields using values for the fitting parameters α, β, τ_s, and τ_φ listed in table S2. Note that fitting with a simple Hikami model (Fig. S2e, dashed line), recovered by assuming $\beta = 0$ in Eq. (2), neglects quasi elastic spin-scattering and fails to describe the data over the entire range of the magnetic field. In the bi-layer sample (Fig. S2f), excellent agreement between fit (black curve) and experimental data is also obtained using Eq. (2), i.e. assuming that the two layers are coupled such that they effectively behave as a single coherent layer. To gain further insight into the electronic coupling in the bilayer, we also present in Fig. S2f theoretical fits (grey curves) to the expression:

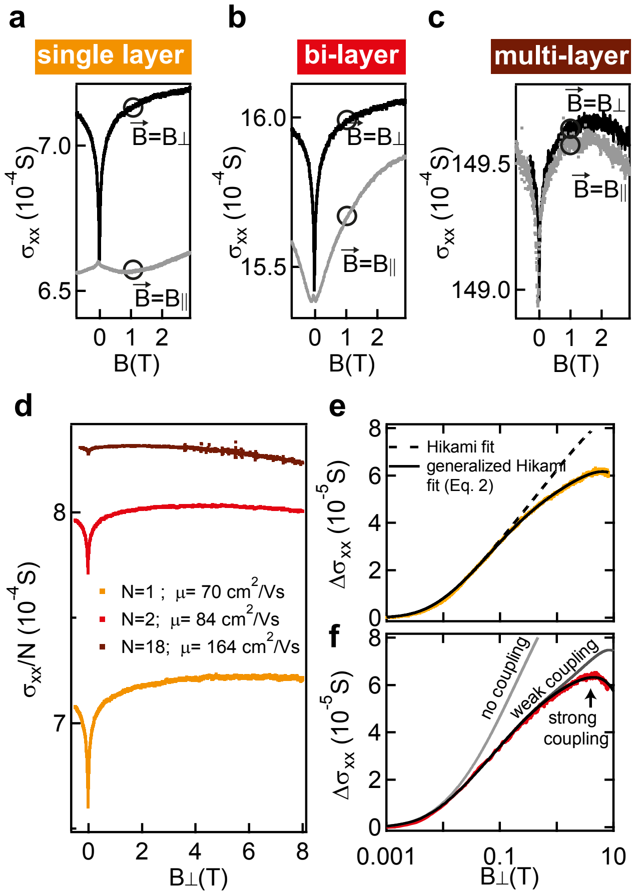

Figure S2: Magnetoconductivity in perpendicular and parallel magnetic field. Conductivity as a function of perpendicular or parallel magnetic field (black and grey lines, respectively) for a (a) single layer, (b) bi-layer, and (c) multi-layer (18 layers) at a temperature of 200 mK. (d) Conductivity per layer as a function of perpendicular magnetic field for the single layer, bi-layer, and multi-layer sample obtained by dividing the black curves in (a), (b), and (c) by 1, 2, and 18, respectively. (e) Weak localization correction to the magnetoconductivity for the single layer sample and theoretical fittings (black curves) obtained with the models described in the text. (f) Weak localization correction to the magnetoconductivity for the bi-layer sample and theoretical fits (grey and black curves) obtained for different electronic coupling regimes. Models are described in the text.

$$\Delta\sigma_{xx}(B_\perp) = \frac{\alpha e^2}{2\pi^2\hbar}\left[F\left(\frac{\hbar}{4eD\tau_\varphi B_\perp}\right) + F\left(\frac{\hbar}{4eDB_\perp}\frac{1+2\frac{\tau_\varphi}{\tau_t}}{\tau_\varphi}\right)\right] - \frac{\beta e^2}{2\pi^2\hbar}F\left(\frac{\hbar}{4eD\tau_s B_\perp}\right) \quad (3)$$

This expression describes tunnel-coupled double quantum wells with a characteristic time τ_s as in Ref 4, and is generalized to include quasi elastic spin scattering as in Eq. (2). For strong tunnel coupling ($\tau_t \leq \tau_e \ll \tau_\varphi$), Eq. (3) reproduces the black curve in Fig S2f, recovering the single layer limit. When the layers are weakly coupled (grey curve in Fig. S2f; $\tau_t/\tau_e = 10$) or fully de-coupled layers (light grey curve in Fig. S2f; $\tau_t/\tau_e = 1000$) the theoretical fits fail to fully reproduce the experimental data. Overall, this quantitative analysis confirms that electron transport in the bi-layer is in the strong coupling regime. As described in the main text, this regime is characterised by the coherent tunnelling of electrons between layers over a timescale comparable to the scattering off dopants within each layer.

	Single layer	Bi-layer
α	1.075 ± 0.004	1.056 ± 0.007
β	0.617 ± 0.001	0.693 ± 0.001
τ_e	0.0097	0.01
τ_φ	25.0 ± 0.2	30.0 ± 0.04
τ_s	0.61 ± 0.01	0.34 ± 0.01
τ_t		≤ 0.01

Table S2. Transport parameters for the single layer and bi-layer sample. τ_e was obtained directly by resistivity and Hall effect measurements. All other parameters were obtained by fitting the weak localisation feature in the magnetoconductance at a temperature of 200 mK.

S3. FITTING PROCEDURE TO EXTRACT LAYER THICKNESS FROM THE ANGULAR DEPENDENCE OF THE WEAK LOCALISATION CORRECTIONS TO THE MAGNETOCONDUCTIVITY

The thickness h of the samples showing two-dimensional character, i.e. the single and bi-layer, is extracted by a quantitative analysis of the angular dependence of the weak localisation corrections to the magnetoconductivity $\Delta\sigma^{WL}(\vartheta) = \sigma(|\vec{B}|, \vartheta)$ measured at $|\vec{B}| = 1$ Tesla. As reported for 2DEGs hosted in other materials systems, such as Si MOSFETs,[5] Si:P delta doped layers,[6] and graphene,[7] the effect of a parallel magnetic field is to enhance the electron dephasing rate by

$$\tau_{\varphi*}^{-1} \rightarrow \tau_{\varphi}^{-1} + \tau_{B_\parallel}^{-1} \tag{4}$$

According to Ref. 5, the additional dephasing rate in parallel magnetic field is given by

$$\tau_{B_\parallel}^{-1} = \left(\frac{e}{\hbar}\right)^2 \frac{\sqrt{\pi} l_e}{\tau_e} Z^2 R B_\parallel^2 \tag{5}$$

where l_e is the mean free path (obtained from resistivity and Hall effect measurements), Z is the root mean square amplitude of the surface describing the two-dimensional electron gas, and R is correlation length over surface height fluctuations. In our angular measurements performed at unitary magnetic field $B_\parallel^2 = sin^2\vartheta$. As in Ref 6, we assume that Z and R correspond to the thickness h of the doping distribution probed electrically and the mean donor spacing within a layer, respectively. R is estimated from the atom probe tomography analysis as $1/\sqrt{n_P}$. We extract h by fitting $\Delta\sigma^{WL}(\vartheta)$ (black lines in Fig. 2a-c) to the phenomenological generalized Hikami-Larkin-Nagaoka expression given in Eq. (2) with $B_\perp = cos\vartheta$ for $|\vec{B}| = 1$ Tesla and using the effective dephasing time $\tau_{\varphi*}$ defined in Eq. (4) instead of τ_φ. Z is used as a single fitting parameter and is linked to the effective dephasing time $\tau_{\varphi*}$. As reported in the main text, we obtain thicknesses of 1.49 ± 0.03 nm and 6.17 ± 0.05 nm in the single and bi-layer sample, respectively. These values are both in agreement with the thickness obtained by atom probe tomography, confirming that the conducting regions in our samples match the extent of the stacked doping profiles.

[†] giordano.scappucci@gmail.com

REFERENCES

[1] Polley, C. M. *et al.* Exploring the Limits of N-Type Ultra-Shallow Junction Formation. *ACS Nano* **7**, 5499–5505 (2013).

[2] Scappucci, G., Capellini, G. & Simmons, M. Y. Influence of encapsulation temperature on Ge:P delta-doped layers.*Phys. Rev. B* **80**, 233202 (2009).

[3] Shamim, S. *et al.* Spontaneous Breaking of Time-Reversal Symmetry in Strongly Interacting Two-Dimensional Electron Layers in Silicon and Germanium. *Phys. Rev. Lett.* **112**, 236602 (2014).

[4] Raichev, O. E. & Vasilopoulos, P. Weak-localization corrections to the conductivity of double quantum wells. *J. Phys.: Condens. Mat.* **12**, 589–600 (2000).
[5] Mathur, H. & Baranger, H. U. Random Berry phase magnetoresistance as a probe of interface roughness in Si MOS-FET's.*Phys. Rev. B* **64**, 235325 (2001).
[6] Sullivan, D. F., Kane, B. E. & Thompson, P. E. Weak localization thickness measurements of Si : P delta-layers. *Appl. Phys. Lett.* **20**, 3814–3824 (2012).
[7] Lundeberg, M. B. & Folk, J. A. Rippled Graphene in an In-Plane Magnetic Field: Effects of a Random Vector Potential. *Phys. Rev. Lett.* **105**, 146804 (2010).
